# What is the most appropriate follow-up time for detecting the epidemiological relationship between coronary artery disease and its main risk factors: novel findings from a 35-year follow-up study

**DOI:** 10.1097/MCA.0000000000001245

**Published:** 2023-05-01

**Authors:** Ari Voutilainen, Christina Brester, Mikko Kolehmainen, Tomi-Pekka Tuomainen

**Affiliations:** a Institute of Public Health and Clinical Nutrition, University of Eastern Finland; b Department of Environmental and Biological Sciences, University of Eastern Finland, Kuopio, Finland

**Keywords:** cohort studies, coronary disease, follow-up studies, men’s health, risk factors

## Abstract

**Background:**

The aim was to investigate the most appropriate follow-up time to detect the associations of coronary artery disease (CAD) with its traditional risk factors in a long-term prospective cohort study.

**Methods:**

The Kuopio Ischaemic Heart Disease Risk Factors Study provided the study material of 1958 middle-aged men free from CAD at baseline and followed up for 35 years. We performed Cox models adjusted for age, family history, diabetes, obesity, hypercholesterolemia, hypertension, smoking, and physical activity, investigated covariate interactions, and tested Schoenfeld residuals to detect time-dependent covariates. Moreover, we applied a sliding window procedure with a subarray of 5 years to better differentiate between risk factors manifested within years and those manifested within decades. The investigated manifestations were CAD and fatal acute myocardial infarction (AMI).

**Results:**

Seven hundred seventeen (36.6%) men had CAD, and 109 (5.6%) men died from AMI. After 10 years of follow-up, diabetes became the strongest predictor of CAD with a fully adjusted hazard ratio (HR) of 2.5–2.8. During the first 5 years, smoking was the strongest predictor (HR 3.0–3.8). When the follow-up time was 8–19 years, hypercholesterolemia predicted CAD with a HR of >2. The associations of CAD with age and diabetes depended on time. Age hypertension was the only statistically significant covariate interaction. The sliding window procedure highlighted the significance of diabetes over the first 20 years and hypertension after that. Regarding AMI, smoking was associated with the highest fully adjusted HR (2.9–10.1) during the first 13 years. The associations of extreme and low physical activity with AMI peaked when the follow-up time was 3–8 years. Diabetes showed its highest HR (2.7–3.7) when the follow-up time was 10–20 years. During the last 16 years, hypertension was the strongest predictor of AMI (HR 3.1–6.4).

**Conclusion:**

The most appropriate follow-up time for most CAD risk factors was 10–20 years. Concerning smoking and hypertension shorter and longer follow-up times could be considered, respectively, particularly when studying fatal AMI. In general, prospective cohort studies of CAD would provide more comprehensive results by reporting point estimates in relation to more than one timepoint and concerning sliding windows.

## Introduction

Diseases follow their unique natural histories including the subclinical stage, the onset of symptoms at the clinical stage, and the chronic stage that may or may not lead to death, and this unique disease progression over time is then modified by living habits and conditions, genetic factors, environmental factors, and treatments [1]. Moreover, the epidemiological relationship between an outcome and a risk factor *per se* weakens over the follow-up, as factors that do not belong to the baseline variables of the research as well as changes in the study participants’ lifestyle, such as quitting smoking, start to affect the risk of the outcome of interest. Consequently, prospective epidemiological studies in general should pay more attention to disease-specific natural histories and their modifiers as well as to probable alterations in baseline characteristics over the follow-up when concluding associations of diseases with their behavior-related risk factors and other exposures [2].

Coronary artery disease (CAD) is caused by atherosclerosis, a plaque buildup in the walls of coronary arteries, and the development of the condition takes years. The speed of progression, however, strongly depends on the clinical manifestation [3]. Coronary arteries can be narrowed up to 70% before symptoms appear and patients suffering from episodes of chest pain, that is, angina pectoris may live with the disease for decades. On the other hand, a plaque may rupture, long before the stenosis produces any symptoms, and lead to an acute myocardial infarction (AMI). Findings from prospective cohorts with exceptionally long follow-ups of up to 50 years suggest the mean follow-up time before the first CAD event of 20 years among middle-aged men [4].

The main risk factors for CAD include age, male sex, and ethnic background together with a family history of CAD that partly reflects certain inherited genetic variants, diabetes, obesity, hypercholesterolemia, hypertension, smoking, and physical inactivity [5]. Like diseases, risk factors have their unique natural histories, as some are effective across the whole lifespan (e.g. genetic variants), some have both immediate and long-term effects (e.g. smoking, and physical activity), and some require years to develop (e.g. obesity). A few meta-analyses on CAD have sub-grouped original studies according to follow-up time and found prehypertension to increase the risk of CAD especially after ≥10 years of follow-up, but no effect of follow-up time on the association of CAD with leisure-time physical activity and impaired fasting glucose [6–9]. A prospective cohort study on nearly 8500 ≥30-year-old Japanese showed variations in the duration of follow-up that most evidently detect the epidemiological association between a risk factor and CAD mortality [10].

The purpose of this study was to investigate, whether the epidemiologically most appropriate follow-up time for CAD in prospective cohort studies is dependent on the modifiable risk factor studied (diabetes, obesity, hypercholesterolemia, hypertension, smoking, and physical inactivity) and/or on the clinical CAD manifestation including any ischemic heart disease (IHD) and the fatal AMI. The most appropriate follow-up time, in this case, refers to the length of follow-up relating to the largest risk ratio (RR) that indicates the association between CAD and a risk factor.

## Methods

### Study participants

The Kuopio Ischaemic Heart Disease Risk Factors (KIHD) Study (Ethical Approval by the Ethical Committee of the Kuopio University on 1 December 1983, the date acting as the reference number) provided the study material [11]. The KIHD recruitment was not completely random but targeted four different age groups, 42, 48, 54, and 60. The participation rate was 82.9%. We included 1958 men (73.0% of those who participated), who underwent KIHD baseline examinations between 27 March 1984, and 14 December 1989, gave written informed consent, and were free from CAD.

The KIHD baseline examinations included a maximal symptom-limited exercise tolerance test. Bicycle ergometers with a linear (Medical Fitness Equipment 400 L, Mearn, the Netherlands) or a step-by-step increase in the workload by 20 W per minute (Tunturi EL 400, Turku, Finland) served as devices for the assessment of physical work. Measurements of oxygen uptake were based on a breath-by-breath method (MGC 2001, Medical Graphics, St. Paul, Minnesota, USA) or a mixing-chamber method (Mijnhardt Oxycon 4, Odijk, the Netherlands). The test procedure consisted of standard 12-lead ECG recordings (Kone, Turku, Finland) before, during, and after the ergometer test. The study participants also answered questions concerning CAD. The criteria for CAD at baseline were as follows: unable to complete the ergometer test due to angina pectoris-type chest pain or Q waves on the ECG indicating a myocardial infarction or horizontal or downsloping ST depression ≥1 mm in aVF or V5 leads or answering ‘yes’ to at least one of the CAD-related questions.

### Coronary heart disease manifestations

This study applied two different manifestations. The first manifestation, CAD as any IHD, referred to International Classification of Diseases (ICD) 10 codes of I20–I25 assigned during hospitalizations (Finnish Institute for Health and Welfare, Care Register for Health Care, Data Permission THL/93/5.05.00/2013). The second manifestation, fatal AMI, referred to ICD 10 codes of I21 as the underlying cause of death (Statistics Finland, Causes of Death, Data Permission TK/782/07.03.00/2021). We gathered data from the national registers, Care Register for Health Care and Causes of Death, until 31 December 2019. There were no losses to follow-up.

### Covariates

This study focused on the main nonmodifiable and modifiable risk factors of CAD as covariates. Age and a family history of CAD were the only nonmodifiable risk factors considered. The family included parents and siblings. Our previous studies explain the KIHD baseline measurement protocol in detail [12,13]. Briefly, study participants gave blood samples (Terumo Venoject VT-100PZ, Terumo Corp., Tokyo, Japan) between 8 and 10 a.m. Before that they had abstained from alcohol for 3 days, smoking and eating for 12 h. In addition to giving blood samples, study participants answered questions concerning the presence of diet-controlled diabetes, the use of medication, smoking habits, and physical activity. The physical activity questions concerned typical 24 h.

Fasting blood glucose (FBG) levels <5.6 mmol/l measured by a glucose dehydrogenase method (Merck, Darmstadt, Federal Republic of Germany) indicated normoglycemia, levels 5.6–6.9 mmol/l prediabetes, and levels >6.9 mmol/l or the use of glucose-lowering medication indicated diabetes. The ratio of weight in kilograms to the square of height in meters, that is, the body mass index (BMI), distinguished normal weight (BMI < 25.0 kg/m^2^) from overweight (25–29.9 kg/m^2^) and obese men (≥30.0 kg/m^2^). The fasting serum LDL-cholesterol (S-LDL-C) concentration served as an indicator of hypercholesterolemia. S-LDL-C < 3.4 mmol/l indicated normal cholesterol, 3.4–4.1 mmol/l borderline high cholesterol, and >4.1 mmol/l or the use of cholesterol-lowering medication high cholesterol. Mean values of systolic (SBP) and diastolic blood pressure (DBP) measured by a sphygmomanometer six times after a rest of 30 min when sitting, standing, and lying down recognized normotensive (SBP < 120 and DBP < 80 mmHg), prehypertensive (SBP 120–139 or DBP 80–89 mmHg), and hypertensive subjects [SBP > 139 or DBP > 89 mmHg or the use of high blood pressure (BP) medication]. We classified study participants into never-smokers, previous regular smokers, and current regular smokers. Regular and current, in this case, means every day or nearly every day for at least 12 months and within the past 30 days, respectively. The unit of smoking was the number of packs smoked per day times years of smoking, that is, a pack-year. We also calculated the total physical activity (TPA) by subtracting the basal energy expenditure (BEE) from the total energy expenditure (TEE) and the physical activity level (PAL) by dividing TEE by BEE.

### Statistical analysis

To detect associations of CAD with its risk factors, we performed a Cox proportional hazards model [14] separately for each manifestation, any IHD and fatal AMI, and adjusted every model for all covariates (age, family history, FBG, BMI, S-LDL-C, BP, smoking, and PAL).

For the most frequent manifestation, any IHD, we investigated all possible multiplicative covariate interactions with Bonferroni corrected *P* values on account of multiple testing as well as the proportional hazards assumption by examining the Schoenfeld residuals [15]. Moreover, to differentiate between risk factors manifested within years after baseline and those manifested within decades, we applied a sliding window procedure with a subarray of 5 years in addition to analyses based on cumulative data.

The IBM SPSS Statistics 27.0.1 (The International Business Machines Corporation, Armonk, New York, USA) and GraphPad Prism 5.03 (GraphPad Software, Boston, Massachusetts, USA) served as statistical platforms for the analyses.

## Results

### Baseline characteristics

Study participants were 42–61 years old men with a prevalence of any family CAD history of 45.4% (father’s CAD 30.0%, mother’s CAD 16.8%, sibling’s CAD 16.5%, missing information 0.8%). Their mean (SD) characteristics related to modifiable CAD risk factors were as follows: FBG 4.7 (1.0) mmol/l, weight 80.5 (12.1) kg, height 173.2 (6.1) cm, BMI 26.8 (3.6) kg/m^2^, serum total cholesterol (STC) 5.9 (1.1) mmol/l, S-LDL-C 4.0 (1.0) mmol/l, SBP 134.4 (16.7) mmHg, DBP 89.2 (10.6) mmHg, smoking exposure 17.5 (16.1) pack-years in previous and 27.1 (18.9) pack-years in current regular smokers, and the TPA 2367.3 (910.1) kcal/day (Table 1). They distributed into the CAD risk factor severity categories as follows: FBG (91.9% no risk, 5.2% moderate risk, 2.9% high risk, 0% missing information), BMI (31.8%, 50.7%, 17.1%, 0.5%), S-LDL-C (27.5%, 29.0%, 41.7%, 1.8%), BP (10.5%, 32.9%, 56.1%, 0.5%), and smoking (34.3%, 34.5%, 31.2%, 0%). Regarding the TPA no men belonged to the high-risk category.

**Table 1:** Baseline characteristics by coronary artery disease status at follow-up

Characteristic	Total	No CAD	CAD	*P* value
*n*	1958	1208	750	N/A
Age (years, mean ± SD)	52.6 ± 5.3	52.0 ± 5.5	53.6 ± 4.6	<0.001
Close relatives with CAD (mean ± SD)	0.6 ± 0.7	0.5 ± 0.7	0.7 (0.8)	<0.001
No close relatives with CAD (*n*, %)	1061 (54.2)	704 (58.3)	357 (47.6)	
One close relative with CAD (*n*, %)	638 (32.6)	371 (30.7)	267 (35.6)	
Two close relatives with CAD (*n*, %)	213 (10.9)	111 (9.2)	102 (13.6)	
Three close relatives with CAD (*n*, %)	31 (1.6)	12 (1.0)	19 (2.5)	
Missing information (*n*, %)	15 (0.8)	10 (0.8)	5 (0.7)	
FBG (mmol/l, mean ± SD)	4.7 ± 1.0	4.7 ± 0.9	4.8 ± 1.1	<0.001
Normoglycemia (FBG < 5.6, *n*, %)	1800 (91.9)	1133 (93.8)	667 (88.9)	
Prediabetes (*n*, %)	102 (5.2)	52 (4.3)	50 (6.7)	
Diabetes (FBG > 6.9, *n*, %)	56 (2.9)	23 (1.9)	33 (4.4)	
BMI (kg/m^2^, mean ± SD)	26.8 ± 3.6	26.6 ± 3.4	27.3 ± 3.7	<0.001
Normal weight (BMI < 25, *n*, %)	622 (31.8)	425 (35.2)	197 (26.3)	
Overweight (*n*, %)	993 (50.7)	597 (49.4)	396 (52.8)	
Obesity (BMI ≥ 30, *n*, %)	334 (17.1)	178 (14.7)	156 (20.8)	
Missing information (*n*, %)	9 (0.5)	8 (0.7)	1 (0.1)	
S-LDL-C (mmol/l, mean ± SD)	4.0 ± 1.0	3.9 ± 1.0	4.2 ± 1.0	<0.001
Normal cholesterol (S-LDL-C < 3.4, *n*, %)	539 (27.5)	390 (32.3)	149 (19.9)	
Borderline high cholesterol (*n*, %)	568 (29.0)	354 (29.3)	214 (28.5)	
Hypercholesterolemia (S-LDL-C > 4.1, *n*, %)	816 (41.7)	440 (36.4)	376 (50.1)	
Missing information (*n*, %)	35 (1.8)	24 (2.0)	11 (1.5)	
SBP (mmHg, mean ± SD)	134.4 ± 16.7	133.0 ± 16.5	136.5 ± 16.7	<0.001
DBP (mmHg, mean ± SD)	89.2 ± 10.6	88.4 ± 10.7	90.6 ± 10.2	<0.001
Normotension (SBP < 120 and DBP < 80, *n*, %)	205 (10.5)	147 (12.2)	58 (7.7)	
Prehypertension (*n*, %)	645 (32.9)	434 (35.9)	211 (28.1)	
Hypertension (SBP > 139 or DBP > 89, *n*, %)	1098 (56.1)	620 (51.3)	478 (63.7)	
Missing information (*n*, %)	10 (0.5)	7 (0.6)	3 (0.4)	
Pack-years (packs/day × years smoking)	14.5 ± 18.0	13.8 ± 17.7	15.6 ± 18.5	0.033
Never smoked regularly (*n*, %)	672 (34.3)	437 (36.2)	235 (31.3)	
Previous regular smoker (*n*, %)	675 (34.5)	403 (33.4)	272 (36.3)	
Current regular smoker (*n*, %)	611 (31.2)	368 (30.5)	243 (32.4)	
TEE (kcal/day)	3996 (968)	3974 (946)	4031 (1001)	0.201
BEE (kcal/day)	1630 (149)	1632 (149)	1627 (149)	0.498
Moderately active (PAL < 2.0, *n*, %)	385 (19.7)	237 (19.6)	148 (19.7)	
Vigorously active (*n*, %)	665 (34.0)	424 (35.1)	241 (32.1)	
Extremely active (PAL > 2.4, *n*, %)	888 (45.4)	534 (44.2)	354 (47.2)	
Missing information (*n*, %)	20 (1.0)	13 (1.1)	7 (0.9)	

CAD, including those who died from acute myocardial infarction; *P* value is from the Mann–Whitney *U*-test or *t*-test for the difference between no CAD and CAD groups.

BEE, basal energy expenditure; BMI, body mass index; CAD, coronary artery disease; DBP, diastolic blood pressure; FBG, fasting blood glucose; PAL, physical activity level as TEE divided by BEE; S-LDL-C, serum LDL-cholesterol; SBP, systolic blood pressure; TEE, total energy expenditure.

### Coronary artery disease

**Fig. 1:**
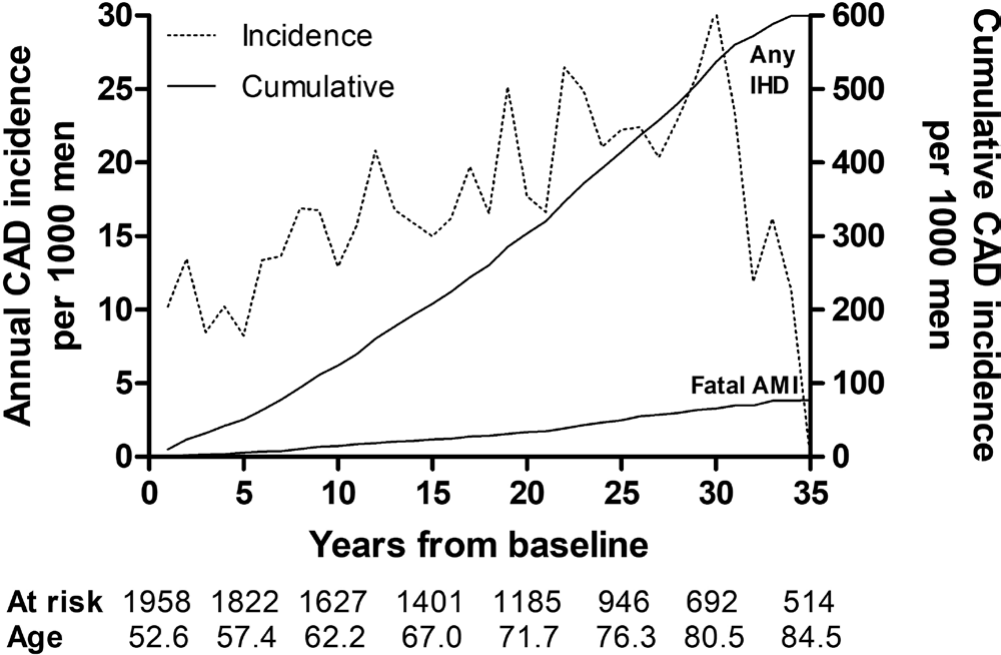
Coronary artery disease (CAD) incidence, as any ischemic heart disease (IHD) and fatal acute myocardial infarction (AMI), in a cohort of 1958 middle-aged eastern Finnish men by the end of 2019.

The cumulative CAD incidence after 35 years of follow-up was 600 per 1000 men (Fig. 1). The annual CAD incidence increased rather linearly for the first 30 years until the mean age of men at risk was 80.5 years. The highest annual CAD incidence was 30 per 1000 men. The mean (SD) follow-up time before the first CAD diagnosis was 15.7 (8.9) years.

**Fig. 2:**
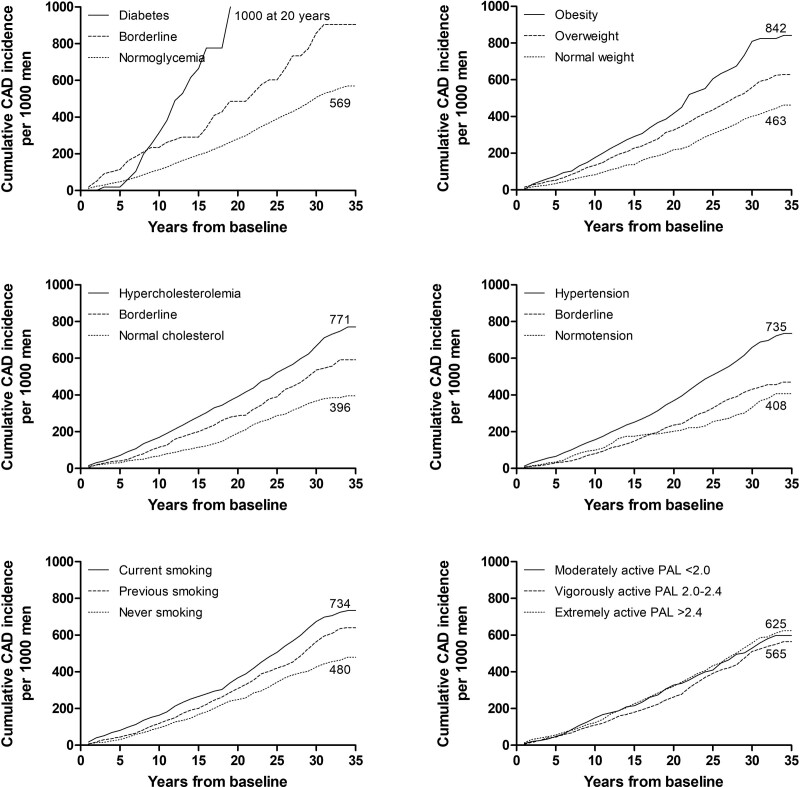
Cumulative coronary artery disease (CAD) incidence by risk factor severity categories in a cohort of 1958 middle-aged eastern Finnish men by the end of 2019.

With respect to modifiable risk factors, men with diabetes showed the highest cumulative CAD incidence (Fig. 2). After 20 years of follow-up all of them had become CAD cases. The second highest cumulative CAD incidence, 842 per 1000 men within 35 years, was related to obesity. Also, hypercholesterolemia, hypertension, and smoking were related to an increased cumulative CAD incidence, whereas normocholesterolemia and normotension resulted in the lowest cumulative CAD incidences, c. 400 per 1000 men.

**Fig. 3:**
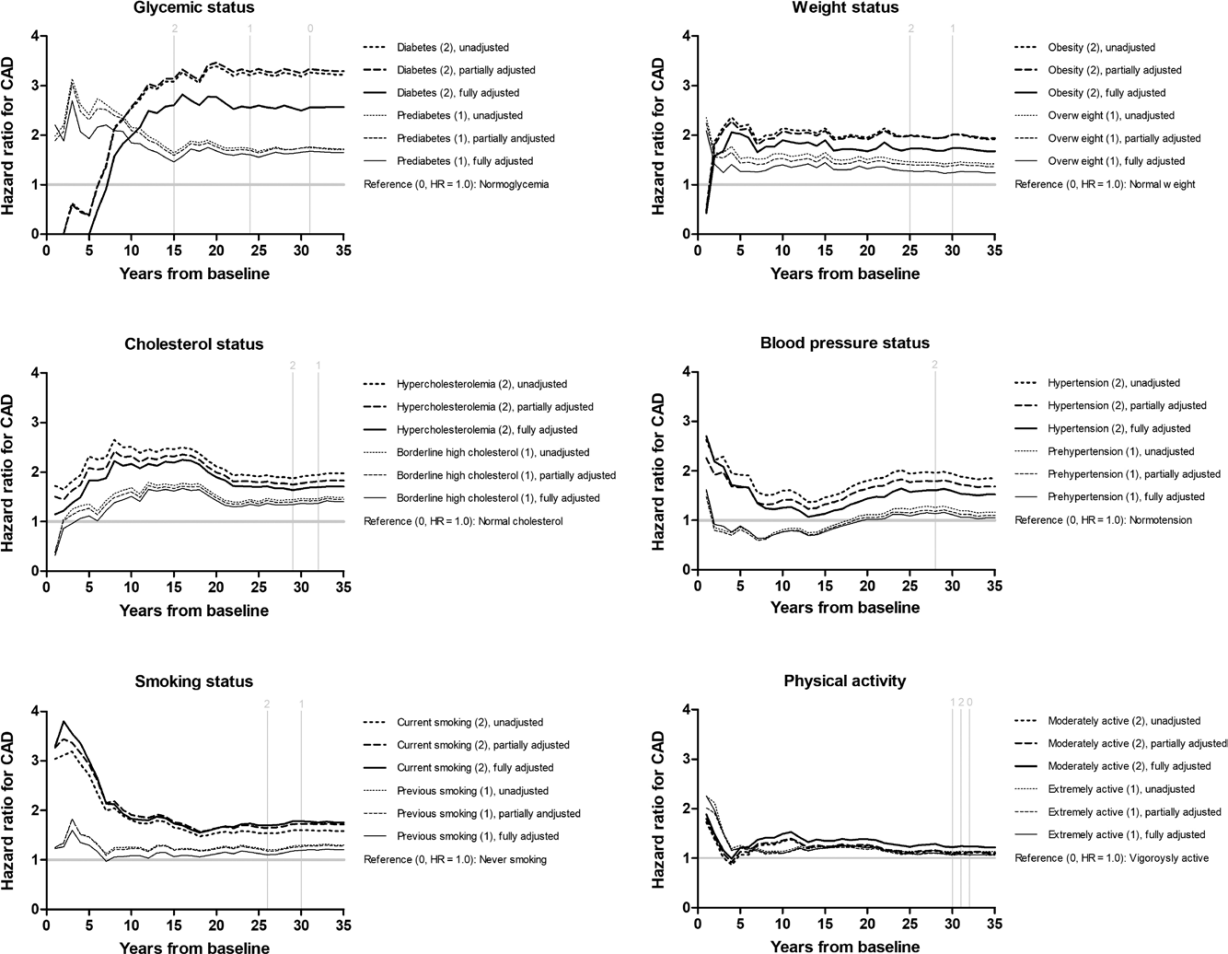
Unadjusted and adjusted hazard ratios for coronary artery disease (CAD) by modifiable risk factors in a cohort of 1958 middle-aged eastern Finnish men by the end of 2019. Vertical lines indicate when the cumulative number of cases exceeded the number still at risk in the reference (0), moderate risk (1), and severe risk categories (2).

The follow-up time related to the most statistically significant relationship between the CAD risk and a risk factor greatly differed across the risk factors (Fig. 3). The survival association of glycemic status with the risk of CAD stabilized not until after the first 15 years of follow-up, whereas the association of weight status with the risk of CAD remained nearly constant throughout the entire follow-up period. The association of cholesterol status with the risk of CAD resulted in its highest hazard ratios (HRs) between 5 and 20 years of follow-up corresponding to the same timespan when the association of BP status with the risk of CAD resulted in its lowest HRs. The association of smoking status with the risk of CAD for its part related specifically to the first 5 years of follow-up.

**Fig. 4:**
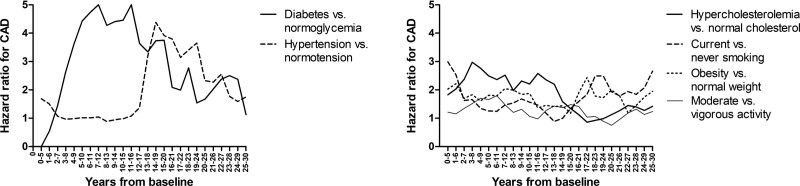
Adjusted hazard ratios for coronary artery disease (CAD) by modifiable risk factors with a sliding window of 5 years in a cohort of 1958 middle-aged eastern Finnish men by the end of 2019.

The sliding window procedure highlighted diabetes and hypertension as the most significant risk factors for CAD. Diabetes increased the risk of CAD particularly over the first 20 years of follow-up, whereas the association of hypertension with the risk of CAD manifested not until 12–13 years after baseline but then stayed evident for almost 10 years (Fig. 4). Smoking, hypercholesterolemia, obesity, and PALs associated with much lower HRs compared to diabetes and hypertension.

On the basis of Schoenfeld residuals the associations of age and glycemic status with the risk of CAD were to some extent time-dependent during the entire follow-up period (Supplementary Figure 1, Supplemental digital content 1, http://links.lww.com/MCA/A563). The association of smoking status with the risk of CAD showed time dependency, specifically, when the follow-up time exceeded 15 years, whereas the association of weight status with the risk of CAD indicated time dependency when the follow-up time was less than 8 years.

Out of 28 possible covariate interactions, only the interaction between age and BP showed statistical significance when the *P* value was Bonferroni corrected on account of multiple testing (Supplementary Figure 2, Supplemental digital content 2, http://links.lww.com/MCA/A564). This interaction was valid after the first 20 years of follow-up.

### Fatal acute myocardial infarction

**Fig. 5:**
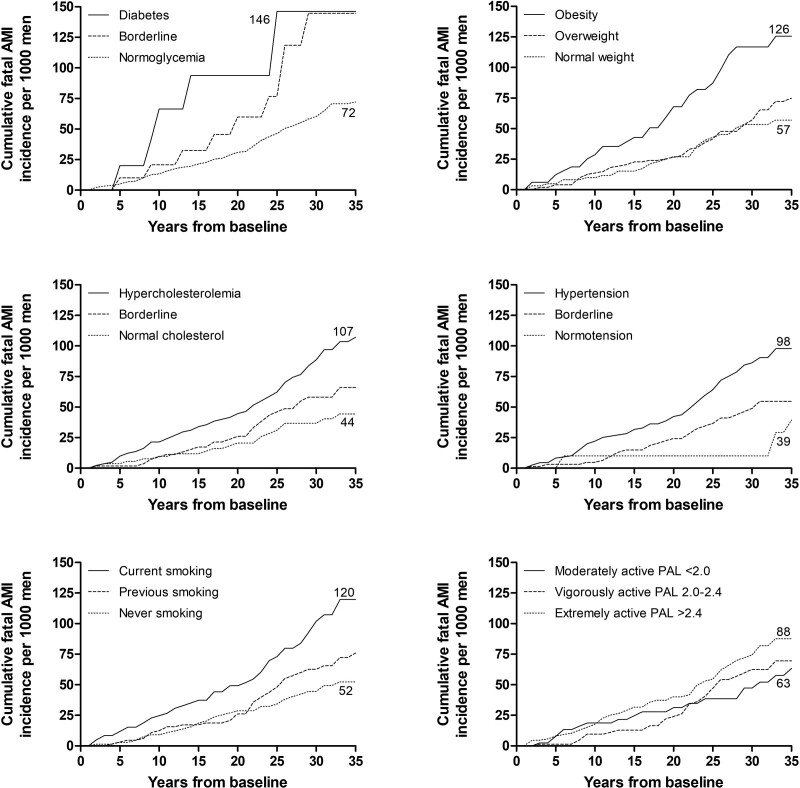
Cumulative fatal acute myocardial infarction (AMI) incidence by risk factor severity categories in a cohort of 1958 middle-aged eastern Finnish men by the end of 2019.

The cumulative fatal AMI incidence was 77 per 1000 men (Fig. 1). The annual incidence ranged from 0 to 6. The mean (SD) follow-up time before the fatal AMI event was 17.8 (9.1) years. Regarding modifiable risk factors the highest cumulative AMI incidence was related to diabetes followed by obesity and smoking (Fig. 5). Also, hypercholesterolemia was evidently associated with an increased AMI incidence. Normotension resulted in the lowest cumulative AMI incidence being extremely low, 10 per 1000 men, over the first 32 years of follow-up. The physical activity showed no clear association with cumulative AMI incidence.

**Fig. 6:**
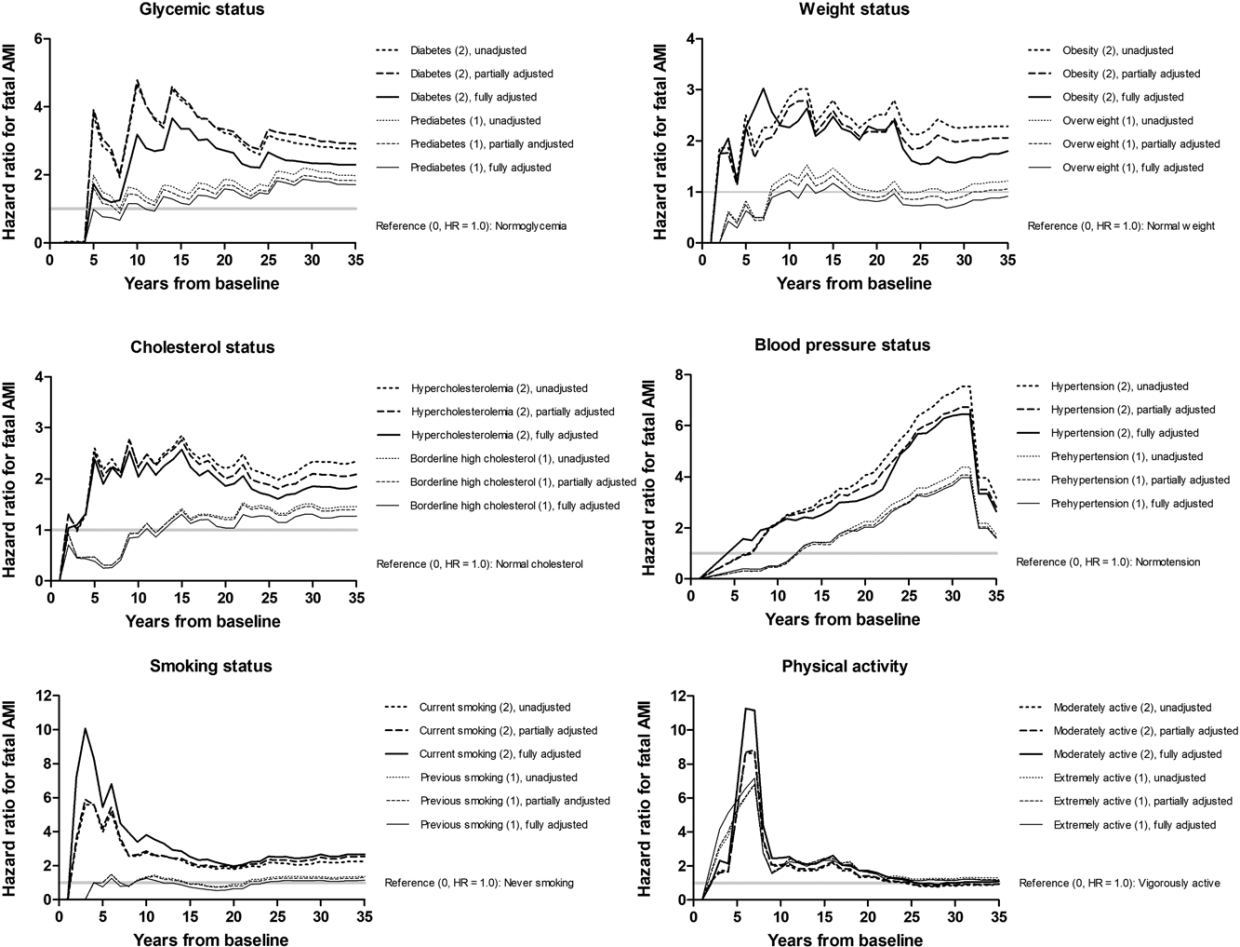
Unadjusted and adjusted hazard ratios for fatal acute myocardial infarction (AMI) by modifiable risk factors in a cohort of 1958 middle-aged eastern Finnish men by the end of 2019 in the reference (0), moderate risk (1), and severe risk categories (2). Note the different y-axes.

The follow-up time did not evidently relate to the survival associations of diabetes, obesity, and hypercholesterolemia with the risk of fatal AMI (Fig. 6). On the contrary, the associations of hypertension and prehypertension with the risk of AMI became stronger when the follow-up proceeded, and the associations of smoking status and physical activity with the risk of AMI peaked during the first 10 years of follow-up. Both moderate and extreme PALs increased the risk of AMI compared to vigorous physical activity.

## Discussion

### Summary of key findings

In the KIHD cohort, the RR for CAD was dependent on the follow-up time irrespective of the manifestation and risk factor, except for obesity which related to a somewhat constant RR of 2 during the entire follow-up period. The association of smoking with the risk of CAD peaked over the first 10 years of follow-up, whereas hypercholesterolemia related to its highest effect sizes during the first 5 years and when the follow-up time exceeded 20 years. The main differences between the manifestations related to hypertension and physical activity. The RR for fatal AMI among hypertensive versus normotensive men increased linearly over the entire follow-up period, whereas the corresponding RR for CAD suggested an S-shaped curve. The RR for fatal AMI among moderately and extremely active men compared to vigorously active men peaked between 5 and 10 years of follow-up, whereas the risk of CAD did not associate with physical activity. Short follow-up times of <15 years appeared to be related to a weak association of diabetes with the risk of CAD but this finding most probably originates from the low number of men with diabetes in the KIHD cohort at baseline. The sliding window procedure emphasized the importance of reporting results at more than one timepoints, as diabetes increased the risk of CAD, particularly during the first 20 years, and hypertension not until after 12 years of follow-up.

### Comparison with previous studies

To the best of our knowledge, there are no previous individual-level within-cohort findings on the relationship between the risk of CAD and follow-up time, except for the study of CAD mortality in NIPPON DATA80 by Okami *et al* [10]. In the NIPPON DATA80 cohort of 3745 men with a follow-up of 29 years, the strongest predictor of CAD mortality during the first 20 years or so was current smoking with an HR of around 3. After that diabetes became the strongest predictor until the end of follow-up with the HR ranging from 2.0 to 2.8. STC was a statistically significant predictor of CAD mortality over the entire follow-up period, but the HR stayed rather low with 1.9 being its maximum. SBP was a statistically significant predictor of CAD mortality only during the last 5 years of follow-up. By and large, the results reported by Okami *et al*. [10] are in accordance with our findings, as smoking was a strong predictor of CAD in the first years of follow-up, then diabetes became the leading predictor, and the role played by hypertension was meaningful at the end of follow-up and specifically in relation to mortality. Moreover, the association of CAD with hypercholesterolemia was moderate, statistically significant, and somewhat independent of the length of follow-up both in the NIPPON DATA80 and KIHD cohorts.

Due to a lack of within-cohort results concerning the survival association between the risk of CAD and follow-up time, the comparison of our individual-level findings with previous studies presented in the following paragraphs is with study-level findings from published meta-analyses. With respect to diabetes, meta-analyses suggested the RR for CAD around two among diabetic men compared with normoglycemic men [16,17]. These meta-analyses combined fatal and nonfatal CADs and did not adjust for follow-up time. The older one of these meta-analyses published in 2000 combined 10 primary studies in which the follow-up time was 4–7 years in five studies, 9 years in two studies, 12–20 years in two studies, and 36 years in one study [16]. The average RR for CAD was 1.5 in the studies with the shortest follow-up time, 3.1 in the studies with the medium follow-up time, and 2.1 in the studies with the longest follow-up time. Correspondingly, the newer one of these meta-analyses published in 2014 combined 21 studies in which the follow-up time ranged from 5 to 25 years [17]. A scatter plot using data from those 21 studies did not reveal any association between the RR for CAD and the follow-up time, and the correlation between them was slightly positive but far from statistical significance (*r* = 0.181, *P* = 0.433). To summarize, both our present individual-level findings and study-level findings based on published meta-analyses suggest that follow-up times of up to 5 years are perhaps too short to be appropriate for detecting the survival association between the risk of CAD and diabetes.

One meta-analysis on the epidemiological relationship between the risk of CAD and obesity combined 17 primary studies and resulted in a pooled HR of 1.6 [18]. The meta-analysis did not adjust for follow-up time or distinguish between fatal and nonfatal CADs. In the 17 primary studies, the HR for CAD did not correlate with the follow-up time (*r* = −0.177, *P* = 0.497) that ranged from 7 to 44 years. This is in accordance with our finding that the strength of the survival association between the risk of CAD and obesity does not depend on the follow-up time *per se*.

The meta-analysis of the association between hypercholesterolemia and CAD published in 2004 by Anum and Adera grouped the primary studies according to the CAD manifestation and age [19]. Regarding CAD incidence, the number of primary studies was three for men followed from middle age and above and six for men followed from age >65. In both these subgroups, the correlation between the RR for CAD and the follow-up time was negative but statistically nonsignificant (*r* = −0.607, *P* = 0.585 and *r* = −0.417, *P* = 0.411). Regarding CAD mortality, the number of primary cohorts for men followed from middle age and above was nine, and the correlation between the RR for CAD and the follow-up time was statistically nonsignificant (*r* = −0.030, *P* = 0.939). The meta-analysis published in 2016 by Peters *et al*. combined 18 primary prospective studies of the association between a 1 mmol/l increase in TC and the incident CAD but adjusted for the duration of follow-up only with respect to the women-to-men ratio of RR for CAD [20]. In this meta-analysis, the follow-up time ranged from 7 to 35 years, and it did not correlate statistically significantly with the RR for CAD (*r* = 0.347, *P* = 0.158). In summary, the study-level findings from published meta-analyses [19,20] or the individual-level findings from the KIHD cohort did not suggest any obvious recommendations concerning the most appropriate follow-up time for investigations of the relationship between CAD and hypercholesterolemia.

A few meta-analyses studied the risk of CAD in prehypertensive and white-coat hypertensive participants, but we did not find meta-analyses reporting the RR for CAD among hypertensive versus normotensive men. Allen *et al*. pooled seven US cohort studies to estimate the lifetime risk for CAD and concluded the risk of around 40% in 55-year-old hypertensive men [21]. Among normotensive men, the corresponding risk was around 25% [21]. Compared to the KIHD cohort, the risks reported by Allen *et al*. are notably lower. Among hypertensive middle-aged male KIHD study participants, the cumulative risk of CAD is 74% and in normotensive peers, it is 41%. The difference in the cumulative risk of CAD between the US and Eastern Finland male populations is expected, as the prevalence of CAD, and particularly that of AMI, was extremely high among eastern Finnish men still in the 1980s when the KIHD study was established [11].

With respect to smoking as a CAD risk factor, the meta-analysis by Hackshaw *et al*. pooled results from 26 primary studies and concluded the RR for CAD of 1.5 among men smoking one cigarette per day and 2.0 among men smoking 20 cigarettes per day [22]. In those 26 studies, the follow-up time ranged from three to 50 years, and the RR for CAD in men smoking 20 cigarettes per day versus nonsmokers did not correlate with it (*r* = −0.071, *P* = 0.731). This noncorrelative study-level relationship between the RR for CAD and follow-up time was surprising, as, in the KIHD cohort, the RR for CAD, irrespective of the CAD manifestation, clearly peaked during the first 5 years.

Sofi *et al*. combined the results of 13 primary studies investigating the CAD risk in physically active versus low and inactive men and reported the pooled RR of 0.76 for highly active and 0.93 for moderately active participants [8]. In those 13 studies, the RR for CAD among highly active men showed a statistically nearly significant positive correlation with the follow-up time (*r* = 0.548, *P* = 0.053), which denoted the association between the risk of CAD and physical leisure-time activity was the strongest in studies with the shortest follow-up times. Sofi *et al*. also performed a sex-nonspecific subgroup analysis of 26 primary studies in relation to the follow-up time but found no difference in the pooled RR between <13 and ≥13 years of follow-up [8]. In the KIHD cohort, the relationship between the risk of CAD and leisure-time physical activity was rather weak for IHD over the entire follow-up period but statistically highly significant for the fatal AMI in follow-up times less than 10 years, except for the first couple of years. Moreover, in the KIHD cohort, the lowest risk of AMI is associated with the middle PAL tertile.

To wrap up, our individual-level results based on the KIHD cohort concerning the follow-up time-dependency of the epidemiological relationship of CAD with its risk factors corresponded to study-level findings from meta-analyses regarding diabetes, obesity, and hypercholesterolemia. With respect to physical activity, the correspondence between individual- and study-level findings was weaker at least partly due to different outcomes. In the KIHD cohort, physical activity related to the fatal AMI, but the study-level findings combined nonfatal and fatal events as well as studies reporting broad (any CAD) and narrow CAD manifestations (fatal MI). Concerning smoking as a CAD risk factor, the individual-level results differed from the study-level findings. Both our study using the KIHD cohort and the study by Okami *et al*. using the NIPPON DATA80 cohort [10] highlighted the role of smoking in the first years of follow-up whereas, in the study-level findings, the follow-up time did not affect the epidemiological relationship between CAD and smoking. Overall, the results based on the KIHD cohort and those based on the NIPPON DATA80 cohort closely resembled each other.

### Possible explanations for findings

Diabetes was the strongest risk factor for CAD in the KIHD cohort. Also, the epidemiological relationship of CAD with obesity was evident. Excluding the first years of follow-up the RRs of CAD among men with diabetes and obese men stayed somewhat stable by the end of the follow-up. The low risk of CAD in men with diabetes up to 10 years of follow-up was due to the low numbers of prediabetes and diabetes cases at baseline. Basically, it appears to take approximately 10 years until the increased risk of CAD among middle-aged men with diabetes starts to show up if the cohort is practically free from diabetes at baseline. The pathophysiological mechanism relating CAD to diabetes is still unclear. In any case, tight glycemic control, that is, maintaining normal or nearly normal circulating blood sugar levels does not reduce the risk of cardiovascular events among persons with diabetes [23], but the association between CAD and diabetes probably originates from inflammatory pathways [24].

There was a lack of prospective cohort studies on the relationship between CAD and hypertension. Perhaps the main reason for this is the active treatment of patients with hypertension in general, which directs meta-analyses on high BP medications instead of hypertension *per se*. From the viewpoint of prospective cohort studies, the active treatment of patients with hypertension makes it difficult to distinguish long-term associations of hypertension with the risk of CAD from those of hypertension medications.

Smoking is associated with the strongest risk of CAD during the first years of follow-up, which reflects the role of smoking as one of the main predictors of all-cause mortality [25]. In other words, as smokers, in general, tend to die younger than nonsmokers, the relative effect of smoking on the CAD risk decreases over time. Moreover, smokers may quit smoking during the follow-up, which reduces their CAD risk [26]. In the KIHD cohort, 49% of smokers reported temporary or permanent smoking cessation at their follow-up visits, but only 3% of nonsmokers reported they have begun smoking. The results based on the individual-level data, obviously, differed from those based on the study-level data with respect to smoking as a CAD risk factor. This may partly relate to ecological fallacy, that is, a failure occurring when group-level interpretations are extended to individuals.

In terms of PALs in the KIHD cohort, the results concerning any IHD manifestation differed from the results regarding the fatal AMI manifestation; however, as extreme physical activity, in general, may induce AMI or sudden cardiac death among susceptible persons [27], this difference was even expected. The fatal AMI is not necessarily the first AMI and, in the KIHD cohort, 58% of men who died from AMI had had a nonfatal AMI or unstable angina before the fatal event.

### Strengths and limitations

The KIHD follow-up study is long enough and includes measures of all main CAD risk factors, which enabled us to perform the present analyses. Moreover, the KIHD study includes results of an exercise test and ECG recordings together with a detailed health interview, which made it possible to exclude study participants who were not free from CAD at baseline. This study also has limitations. The present analyses did not include women or physically inactive men, which lowers the generalizability of our results. We also acknowledge competing events may affect the epidemiologically most appropriate follow-up time. In our recent publications, we showed that bias in the Kaplan–Meier estimates increases together with follow-up time [28] and demonstrated the use of a competing risk regression model and cause-specific hazards in the epidemiological relationship of prostate cancer with vitamin D [29]. In this study, we ignored competing events for the sake of clarity and aimed at emphasizing the effect of follow-up time as such.

### Conclusion

The epidemiologically most appropriate follow-up time for most CAD risk factors in the KIHD cohort of middle-aged men was 10–20 years. With respect to smoking, shorter and regarding hypertension, longer follow-up times could be considered, particularly, when studying their associations with fatal AMI. Prospective cohort studies on CAD in general could produce more comprehensive results by taking this dependency on follow-up into account in survival models. Meta-analyses on the epidemiological relationship of CAD with its risk factors also may benefit from adjusting for the study-level follow-up time, notably, concerning diabetes and, to some extent, PALs. The association between the risk of CAD and obesity was least dependent on the follow-up time among the health behavior-related CAD risk factors studied here.

## Acknowledgements

The authors wish to thank the Kuopio Research Institute of Exercise Medicine and the Research Institute of Public Health and Clinical Nutrition of the University of Eastern Finland, Kuopio, Finland, for the data collection.

### Conflicts of interest

There are no conflicts of interest.

## Supplementary Material


