# Supplementary material for: What is the most appropriate follow-up time for detecting the epidemiological relationship between coronary artery disease and its main risk factors: novel findings from a 35-year follow-up study
Source: Coron Artery Dis. 2023 May 1;34(5):320–31. doi: 10.1097/MCA.0000000000001245 (PMC10836792; doi:10.1097/MCA.0000000000001245)

Supplementary Figure 1. P-values for the time dependence of Schoenfeld residuals by risk factors. Horizontal grey lines indicate the p-value of 0.05.

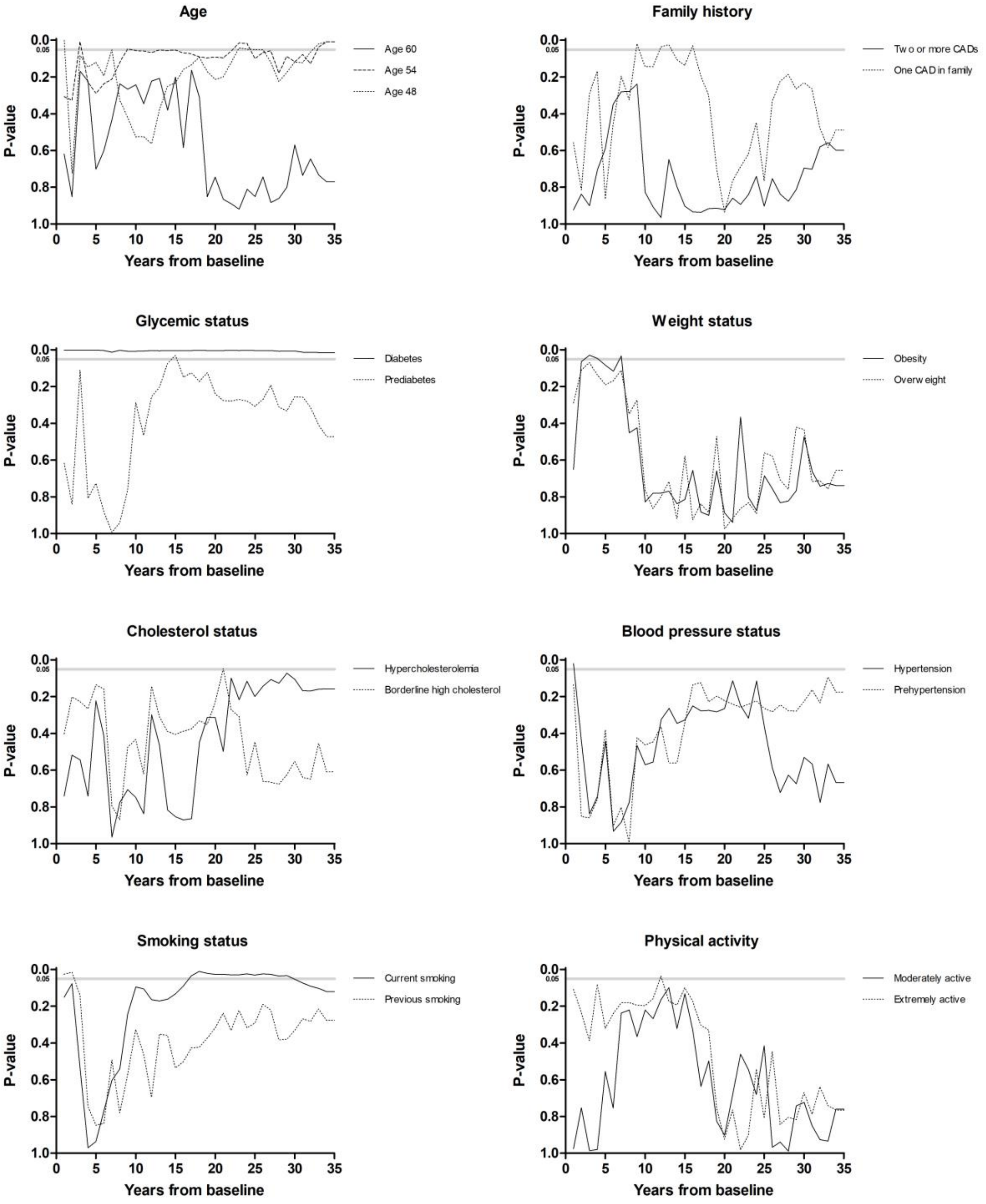

Supplement: Supplementary file 1 [file cad-34-320-s001.pdf]
